# Supplementary material for: Generating semantic maps through multidimensional scaling: linguistic applications and theory
Source: Corpus Linguist Linguist Theory. 2022 Jan 10;18(3):627–65. doi: 10.1515/cllt-2021-0018 (PMC9536326; doi:10.1515/cllt-2021-0018)
Supplement: Supplementary file 1 — Supplementary Material [file j_cllt-2021-0018_suppl.pdf]

Martijn van der Klis* and Jos Tellings

Generating semantic maps through multidimensional scaling: linguistic applications and theory

Supplementary Materials

1 MDS: Mathematical background

In section 2 of the main paper, we described the version of multidimensional scaling (MDS) that is usually known as *classic scaling* or *classic MDS*, or more fully as *classic metric Torgerson scaling*, named after the work of Torgerson (1952). In section 1.1, we detail some other MDS algorithms. Section 1.2 lists some suggestions for further reading.

1.1 Other MDS algorithms

As mentioned in the main paper, classic scaling is only one of various MDS algorithms available. Here we briefly discuss two other algorithms that have been used in the linguistic MDS literature.

A method developed in de Leeuw and Heiser (1977) and subsequent work is based on an iterative method for minimizing a function, known as *majorization.* The corresponding MDS algorithm that minimizes stress by majorization is known as SMACOF (Scaling by MAjorizing a COmplicated Function) (Borg and Groenen 2005, §8; de Leeuw and Mair 2009). In practice, this implementation of MDS is more commonly used than classic scaling, as it is a more flexible and readily available method, for example implemented in R (see Borg, Groenen, and Mair 2018, sections 9 and 10 for a discussion of the benefits and potential issues of SMACOF).

The algorithm referred to as *Optimal Classification* (OC) by Croft and Timm (2013), belongs to a model that is known variously as an *unfolding model*, an *ideal point model*, or a *preference model* (see Borg and Groenen 2005, §14; Ding 2013, p. 247; Groenen and Borg 2014, p. 100; Borg, Groenen, and Mair 2018, §8 for more details).

***Corresponding author: Martijn van der Klis,** UiL OTS, Utrecht University, The Netherlands
Jos Tellings, UiL OTS, Utrecht University, The Netherlands

Unfolding is often classified as a technique closely related to MDS, but distinct from it with respect to the sort of input data. Unfolding is intended to map *preference data*, for example voting behavior of a number of individuals (Poole 2005). Both the individuals and their choice preferences are represented in the output visualization. The linguistic equivalent of this, as explicated by Croft and Poole (2008, §3), is to interpret the possibility to express function f by form s, as a (binary) preference for s by an individual f. Unfolding was mostly used in the earlier linguistic MDS literature, which was interested in the automatic generation of maps that captured the same insights as classical semantic maps, as discussed in section 3.1 of the main paper.

1.2 Further reading

For reasons of space the mathematical introduction in the main paper had to be very concise. For readers who would like to read a more thorough introduction, or are interested in further details, here are some suggestions for further reading:

- A simple online interactive demonstration version of Figure 2 in the main paper can be found at https://demonstrations.wolfram.com/EigenvectorsByHand/
- There are numerous linear algebra textbooks, which introduce both matrices and their operations, and eigenvalues and eigenvectors. An example is:

  Gilbert Strang (2016). *Introduction to Linear Algebra*, 5th edition. Wellesley-Cambridge Press. ISBN 978-0-9802327-7-6.

  See chapter 6 on eigenvalues and eigenvectors (see also http://math.mit.edu/~gs/linearalgebra/).
- For readers who are primarily interested in statistical applications of eigenvector analysis, and methods such as PCA, various online resources, and suitable textbooks are available. See Jolliffe (2005) for a short encyclopedia entry.
- Overview works on MDS (mathematically advanced) that are cited here and in the main paper are Ding (2013, 2018), Borg and Groenen (2005), Groenen and Borg (2014), and Borg, Groenen, and Mair (2018).

2 Dimension interpretation: (Multiple) Correspondence Analysis

In section 3.1 of the main paper, we discuss ways to interpret the dimensions of an MDS map. (Multiple) Correspondence Analysis (CA) is a method related to MDS, and facilitates dimension interpretation through the addition of supplementary points on the map. Whereas MDS takes a dissimilarity matrix as its input, CA uses all two-

way cross-tabulations between categorical variables, and is therefore more suited for a dataset that has been extensively annotated. In cognitive and typological linguistics, CA has been used a.o. to create semantic maps of cutting and breaking events in Majid et al. (2007), the relation between *to bother*, *to annoy*, and *to hassle* in Glynn (2010), causatives in Levshina et al. (2013) and Levshina (2016), and adjectival profiling of shame in Krawczak (2018). Importantly, CA can be supplemented with *passive variables* (i.e., points that are added only after the construction of the map) to see how the resulting dimensions are associated with individual constructions. Such points then assist in the interpretation of the crucial semantic dimensions in the dataset. For example, Levshina (2016) finds lexical causatives (e.g. *to kill*) across languages to end up in similar parts of the map. These passive variables are associated with the functions of direct and intentional causation. On the other hand, analytic causatives (e.g. *to cause to die*) are more widely distributed and thus more semantically diverse, although for most languages these forms relate to indirect causation and autonomous causees (see her Figures 1 and 2).

References

Borg, Ingwer and Patrick J. F. Groenen (2005). *Modern multidimensional scaling: Theory and applications*. Springer Science & Business Media. DOI: 10.1007/0-387-28981-X.

Borg, Ingwer, Patrick J. F. Groenen, and Patrick Mair (2018). *Applied multidimensional scaling and unfolding*. Springer. DOI: 10.1007/978-3-319-73471-2.

Croft, William and Keith T. Poole (2008). “Inferring universals from grammatical variation: Multidimensional scaling for typological analysis”. In: *Theoretical Linguistics* 34.1, pp. 1–37. DOI: 10.1515/THLI.2008.001.

Croft, William and Jason Timm (2013). “Using Optimal Classification for multidimensional scaling analysis of linguistic data”. URL: http://www.unm.edu/~wcroft/MDSfiles/MDSforLinguists-UserGuide.pdf.

de Leeuw, Jan and Willem J. Heiser (1977). “Convergence of correction matrix algorithms for multidimensional scaling”. In: *Geometric representations of relational data*. Ed. by J. C. Lingoes. Mathesis Press, pp. 735–752.

de Leeuw, Jan and Patrick Mair (2009). “Multidimensional scaling using majorization: SMACOF in R”. In: *Journal of Statistical Software* 31.3. DOI: 10.18637/jss.v031.i03.

Ding, Cody S. (2013). “Multidimensional scaling”. In: *The Oxford handbook of quantitative methods in psychology*. Ed. by Todd D. Little. Oxford University Press, pp. 235–256. DOI: 10.1093/oxfordhb/9780199934898.013.0012.

Ding, Cody S. (2018). *Fundamentals of applied multidimensional scaling for educational and psychological research*. Springer. DOI: 10.1007/978-3-319-78172-3.

Glynn, Dylan (2010). “Synonyme, lexical fields, and grammatical constructions. A study in usage-based cognitive semantics”. In: *Cognitive Foundations of Linguistic Usage Patterns*. Ed. by Hans-Jörg Schmid and Susanne Handl. Berlin, New York: De Gruyter Mouton, pp. 89–118. DOI: 10.1515/9783110216035.89.

Groenen, Patrick J. F. and Ingwer Borg (2014). “Past, present, and future of multidimensional scaling”. In: *Visualization and verbalization of data*. Ed. by Jörg Blasius and Michael Greenacre. CRC Press, Taylor & Francis Group, pp. 95–117. DOI: 10.1201/b16741-13.

Jolliffe, Ian T. (2005). “Eigenvalue/Eigenvector”. In: *Encyclopedia of Statistics in Behavioral Science*. Ed. by Brian S. Everitt and David Howell. Wiley, pp. 542–543. DOI: 10.1002/0470013192.bsa189.

Krawczak, Karolina (2018). “Reconstructing social emotions across languages and cultures”. In: *Review of Cognitive Linguistics* 16.2, pp. 455–493. DOI: 10.1075/rcl.00018.kra.

Levshina, Natalia (2016). “Why we need a token-based typology: A case study of analytic and lexical causatives in fifteen European languages”. In: *Folia Linguistica* 50.2, pp. 507–542. DOI: 10.1515/flin-2016-0019.

Levshina, Natalia, Dirk Geeraerts, and Dirk Speelman (2013). “Mapping constructional spaces: A contrastive analysis of English and Dutch analytic causatives”. In: *Linguistics* 51.4, pp. 825–854. DOI: 10.1515/ling-2013-0028.

Majid, Asifa, Melissa Bowerman, Miriam van Staden, and James S. Boster (2007). “The semantic categories of cutting and breaking events: A crosslinguistic perspective”. In: *Cognitive Linguistics* 18.2, pp. 133–152. DOI: 10.1515/COG.2007.005.

Poole, Keith T. (2005). *Spatial models of parliamentary voting*. Cambridge University Press. DOI: 10.1017/CBO9780511614644.

Strang, Gilbert (2016). *Introduction to Linear Algebra*. 5th edition. Wellesley, MA: Wellesley-Cambridge Press.

Torgerson, Warren S. (1952). “Multidimensional scaling: I. Theory and method”. In: *Psychometrika* 17.4, pp. 401–419. DOI: 10.1007/BF02288916.
